# Fast exploration of an optimal path on the multidimensional free energy surface

**DOI:** 10.1371/journal.pone.0177740

**Published:** 2017-05-18

**Authors:** Changjun Chen

**Affiliations:** Biomolecular Physics and Modeling Group, School of Physics, Huazhong University of Science and Technology, Wuhan, Hubei, China; University of Calgary, CANADA

## Abstract

In a reaction, determination of an optimal path with a high reaction rate (or a low free energy barrier) is important for the study of the reaction mechanism. This is a complicated problem that involves lots of degrees of freedom. For simple models, one can build an initial path in the collective variable space by the interpolation method first and then update the whole path constantly in the optimization. However, such interpolation method could be risky in the high dimensional space for large molecules. On the path, steric clashes between neighboring atoms could cause extremely high energy barriers and thus fail the optimization. Moreover, performing simulations for all the snapshots on the path is also time-consuming. In this paper, we build and optimize the path by a growing method on the free energy surface. The method grows a path from the reactant and extends its length in the collective variable space step by step. The growing direction is determined by both the free energy gradient at the end of the path and the direction vector pointing at the product. With fewer snapshots on the path, this strategy can let the path avoid the high energy states in the growing process and save the precious simulation time at each iteration step. Applications show that the presented method is efficient enough to produce optimal paths on either the two-dimensional or the twelve-dimensional free energy surfaces of different small molecules.

## Introduction

In computational biophysics and biochemistry, people are always interested in finding out how the molecule transfers from the reactant state to the product state [1, 2]. From the most optimal path, one can know the detail information about the reaction, like the movements of the different components in the molecule, the direction and the rate of the reaction. Although being important, finding an optimal path is not easy in the simulation. There are three problems should be considered. One is the determination of the possible collective variables. Large number of collective variables could characterize the important states of the molecule precisely. Efficient sampling on the collective variables allows one to study the reaction from the reactant to the product reliably. However, increasing the number of the collective variables also expands the sampling scope in the collective variable space. The sampling speed is also slowed down. So, one must choose the collective variables carefully in the simulation. In theory, good and reasonable collective variables should be independent of each other as much as possible and change smoothly in the conformations between the reactant and the product.

The second is the path construction problem. One must build an initial path first for the subsequent optimization. This seems easy. Since both the reactant state and the product state are characterized by the collective variables, a simple linear or higher order interpolation between the states in the collective variable space can satisfy the requirement [3, 4]. However, in some situations, the interpolated path may pass a lot of high energy states, which cause the further free energy calculation to be unreliable. For example, for a small pentapeptide in Ref. [4], only six straight paths between its seven stable states can be utilized in the free energy calculations. Some paths are just abandoned because of the steric clashes between the atoms. Unlike the pure geometric method, a few years ago we proposed a potential-based method to build a feasible path quickly [5]. By a quasi-Newton optimization method that updates the Hessian matrix iteratively with the limited memory Broyden-Fletcher-Goldfarb-Shanno algorithm (L-BFGS) [6, 7], the molecule in the reactant state is slowly pulled to the product state. All the snapshots sampled in the optimization are connected together to form the complete path. The method is almost as fast as the above interpolation method because no MD simulations are required in the construction of the path [5]. But we must note that, this method only deduces an initial path on the potential energy surface, not the free energy surface. Additional optimization of path on the free energy surface must be performed subsequently.

Compared with the above path construction problem, the path optimization problem is more difficult. This is because that the path connecting the reactant and the product exists in a high dimensional space spanned by the collective variables. It totally has *n*×*m* degrees of freedom. Here *n* and *m* are the number of the collective variables and the snapshots on the path, respectively. Such a large number of degrees of freedom increase the complexity of the system. Improper movements of the snapshots in the collective variable space will cause the path to form kinks. These kinks are fatal to the optimization. In the past, many methods have been proposed to circumvent this issue. They have one common objective: optimizing the energy gradients or the free energy gradients of the snapshots orthogonal to the path as much as possible and remaining the smoothness of the path at the same time. But they use different strategies to reach the objective. In the simulation, some methods try to separate the successive snapshots on the path by additional restrained potentials, like elastic band (EB) [8], nudged elastic band (NEB) [9] and minimum Hamiltonian path (MHP) [10]. Only the normal components of the forces on the snapshots (perpendicular to the path) are used to update the positions of the snapshots in the collective variable space. Other methods try to make the free energy gradients to be parallel to the tangent vectors of the path iteratively, like the string method [11]. String method constructs the initial path in the collective variable space and optimizes its shape by two evolution and reparameterization operations [11–14]. Besides, as two useful variants of the string method, the climbing string method is proposed for searching the transition states [15] and the freezing string method is proposed for decreasing the gradients calculations [16, 17]. Recently, we apply the constrained dynamics method [18–25] in the calculation of the free energy gradients of the snapshots on the path [26] and show that the constrained dynamics method [18–25] is also a useful technique for the path optimize problem [27].

Unlike the above methods, there are other methods that do not use the free energy gradients. For example, string method [11] has a modified version [28]. In the simulation, all the snapshots on the path are updated by the average drifts of swarms of short and unbiased trajectories from their positions in the collective variables space. After a few iterations, the method finally optimizes the initial path to the most probable transition path [28]. Besides, path metadynamics [29] and tomographic method [30, 31] update the path in a more direct way. They calculate the average positions of the snapshots (as in path metadynamics [29]) or the free energy minima (as in tomographic method [30, 31]) on the perpendicular hyperplanes by different strategies and move the snapshots towards the targets subsequently. All the methods discussed above are valid for the optimization of the path. In practice, they can all decrease the free energy barriers on the path effectively. However, these methods require the MD simulations for all the snapshots at every iteration step. Considering so many degrees of freedom in the system, the optimization will be a very time-consuming process.

To save the calculation quantity in the optimization, Chakraborty's group proposed a growing string method in 2004 [32]. This method does not require a guess of the initial path. It builds two path fragments from the reactant and the product simultaneously in the simulation. When the ends of the two path fragments merge to each other, one complete path between the reactant and the product is formed [32]. Since the path is also optimized repeatedly in the growing process and its gradient of the potential is forced to be parallel to the tangent vector of the path, the final path will automatically converge to the minimum energy path (MEP) [32]. Some applications of the method show that it is more efficient than the original string method [33–35]. However, this double-ended growing string method [32] and other related single-ended versions [36, 37] are designed to find the optimal path on the energy surface [32–39]. As far as we know, they have not been applied to the free energy surface till now. As comparison, Ref. [40] provides a method that traces the minimum energy path on the free energy surface. But it requires a prior knowledge of the transition state. It goes downhill on the free energy surface step by step from the transition state to the reactant or the product independently. No further optimization of the path is performed in the simulation [40]. Recently, an anharmonic downward distortion following method (ADDF) is proposed in Ref. [41] for the search of the reaction path from the reactant only. It searches, records and removes the neighboring local minima (called anharmonic downward distortion, ADD) from the reactant successively in the calculation. Combination of the ADDs produces the possible reaction paths and the final products. Ref.[42] performs metadynamic simulation on the path collective variables to search the reaction paths. Unlike the normal expression, it combines the coordination numbers of the atoms with the path collective variables.

In this paper, we use the idea of the single-ended growing string method [36, 37] to build an optimal path on the free energy surface. At the beginning, the path starts from the reactant and extends its length constantly to the product. Different from the growing string method [32], the location of the new snapshot on the path is determined by the free energy gradient (not the energy gradient) of the snapshot and the vector from the snapshot to the product. When each new snapshot is grown on the path, the incomplete path will be optimized in the collective variable space by the constrained dynamics method [18–25]. These two growing and optimizing processes are carried out repeatedly one after the other. They will not be stopped until the end of the path reaches the product.

Compared with the previous full-path optimization methods on the free energy surface, our method has two advantages. First, it does not need an initial path at the beginning. The method starts the path construction from one single state (could be any free energy minimum). Second, it does not have to optimize all the snapshots on the whole path from the reactant to the product at every iteration step. The method reduces the size of the system. This could not only decrease the calculation quantity but also accelerate the convergence of the optimization under certain situations.

It must be noted that this idea has also been used in some other path sampling methods. For example, transition path sampling method (TPS) [43, 44] obtains an ensemble of transition paths by a shooting strategy. It randomly changes the momenta of atoms of the snapshots on the existed path to generate lots of new paths. From the transition probabilities, it is capable of finding the most optimal path. And steered molecular dynamics (SMD) [45, 46] or targeted molecular dynamics (TMD) [47, 48] puts an artificial potential on the molecule at the reactant and pulls it gradually to the product. With proper initial conditions, SMD/TMD can generate an reasonable path [49] or path ensemble [50] between the states. The presented method in this paper has similarities with TPS and SMD/TMD. It can start the optimization from an initial guess path as TPS or from one single state as SMD/TMD. But the optimization is performed in a different way. It lets the free energy gradient determine the growing direction of the path. And in TPS, SMD or TMD, the direction is determined by the potential energy gradient in the standard MD simulation.

## Theoretical methods

To characterize some important states of a molecule and describe its chemical reaction or state transition process, one must use a set of collective variables **(***χ*_1_, *χ*_2_, …, *χ*_*n*_). These collective variables are the functions of the Cartesian coordinates of the atoms, like bonds, angles or dihedrals. How to find a proper collective variable for a transition process is difficult. In general, the values of the collective variables should change greatly in the process. This makes the sampling in the space spanned by the collective variables more efficient than the sampling with the Cartesian coordinates of the atoms. Therefore, it is convenient to define and optimize the transition path in the collective variable space. In the simulation of any snapshot on the path, all the collective variables should be fixed simultaneously and the conformation of the molecule is restricted to a small structural ensemble. This is very critical for the calculation of the averages of the thermodynamic variables in a limited time period. Since these collective variables are only related to a small number of atoms in solute, normally they can be constrained in the simulation by solving the constraint equation [51] at every step, no matter there are other solvents or electrons in the system or not.

Traditionally the optimization should start from a complete path that connects the reactant (represented by a vector **χ**^(*r*)^) and the product (represented by a vector **χ**^(*p*)^). But as we mentioned before, such a complete path is not easy to construct in a high-dimensional space. The more collective variables are in use, the more high energy states could exist on the interpolated path. In the simulation of any snapshot on the path, the molecule has to satisfy many constraint equations with the collective variables. Any conflict of the collective variables may cause the optimization to fail.

Following the idea of the single-ended growing string method on the energy surface [36, 37], here we apply a growing strategy to resolve this issue on the free energy surface. The path is initialized from the reactant state. Then a series of snapshots are grown out of the path one by one with a predefined growth step size *s*_*g*_. Specifically, for a growing path with *i*−1 snapshots, if the distance between the end of the current path (state vector **χ**^(*i*−1)^) and the product (state vector **χ**^(*p*)^) is smaller than the growth step size (|**χ**^(*i*−1)^−**χ**^(*p*)^|<*s*_*g*_), the product state is directly set as the *i*th snapshot on the path (**χ**^(*i*)^ = **χ**^(*p*)^). Then the whole path is built out successively. Otherwise, the *i*th snapshot on the path is determined by the growing direction vector **P**(*i*) *g* with a step size *s*_*g*_

$$\chi^{\left(i\right)}=\chi^{\left(i-1\right)}+s_{g}{\text{P}}_{g}^{\left(i\right)}$$ (1)

Here the growing direction vector **P**(*i*) *g* is calculated by

$${\text{P}}_{g}^{{}^{\left(i\right)}}=-\nabla F(\chi^{\left(i-1\right)})+w\cdot \frac{\chi^{\left(p\right)}-\chi^{\left(i-1\right)}}{\left|\chi^{\left(p\right)}-\chi^{\left(i-1\right)}\right|}$$ (2)

In the formula, ∇*F*(**χ**^(*i*−1)^) is the derivative of the free energy function *F* with respect to the collective variables of the (*i*−1)th snapshot (free energy gradient or thermodynamic mean force). *w* is the weighting factor, which determines the curvature of the path. The determination of the parameters in the equations depends on our demands on the final path. If one wants to insert more snapshots to the path, the growth step size *s*_*g*_ should be small. If one wants to make the path more flexible so that it can pass more intermediate states, the weighting factor *w* should be small too.

When a new snapshot is grown, we do a similar reparametrization step as in the string method [12]. The length of the current path in the collective variable space is calculated by Simpson's rule and then the path is divided into pieces evenly according to the number of the snapshots. Then all the snapshots are moved to their new locations on the same path by SHAKE algorithm [51]. To be distinguished from the interpolation method, from now on, this path construction method will be called the growing method.

In the growing process, the free energy barrier on the path is required to be decreased. One can use the formula of the string method [11]. It is derived from the condition of the minimum energy path (MEP) in the Cartesian coordinate space [11]. By some transformations from the Cartesian coordinates to the collective variables, it gives the following optimization formula on the free energy surface (see Eq. (46) in Ref. [11])

$$\chi_{new}^{\left(i\right)}=\chi_{old}^{\left(i\right)}+s_{op}{\text{P}}_{op}^{\left(i\right)}$$ (3)

$${\text{P}}_{op}^{\left(i\right)}=-\text{M}\cdot \nabla F(\chi^{\left(i\right)})+(\text{M}\cdot \nabla F(\chi^{\left(i\right)})\cdot {\text{s}}^{\left(i\right)}){\text{s}}^{\left(i\right)}$$ (4)

Here **χ**(*i*) *old* and **χ**(*i*) *new* are the current and the new position of the *i*th snapshot in the collective variable space, respectively. *s*_*op*_ is the step size. **P**(*i*) *op* is the optimization direction for the *i*th snapshot, which depends on three quantities: the unit tangent vector of the path **s**^(*i*)^, the free energy gradient ∇*F*(**χ**^(*i*)^) and the metric tensor matrix **M**. The first quantity can be easily determined by the interpolation on the positions of the snapshots in the collective variable space. The second quantity is the derived result from the free energy calculation. We will discuss this issue later. The last quantity, metric tensor **M**, contains the averages of the derivatives of the collective variables with respect to the related Cartesian coordinates in the simulation. Briefly speaking, the formula in the string method requires the projected free energy gradient **M**·∇*F* to be parallel to the tangent of the path in the collective variable space [11].

The above formula works for the path optimization. But in this work, we want to compare the results with the optimization on the complete free energy surface. Generally the surface is constructed by the efficient full-space sampling methods like Metadynamics [52–55] or adaptively biased molecular dynamics (ABMD) [56–58]. On the existed surface, an optimal path can also be obtained. But in the construction of the complete free energy surface, it is difficult to perform the sampling in the whole collective variable space by the repulsive biasing potentials and the calculation of the metric tensor **M** at the same time. This is because the Hamiltonian of the system is modified by the biasing potentials. To make a comparison between the optimizations, we assume the metric tensor **M** to be diagonal [27]

$${\text{P}}_{op}^{\left(i\right)}=-\nabla F(\chi^{\left(i\right)})+(\nabla F(\chi^{\left(i\right)})\cdot {\text{s}}^{\left(i\right)}){\text{s}}^{\left(i\right)}$$ (5)

So it forces the free energy gradient ∇*F* to be parallel to the tangent of the path (not **M**·∇*F*). Actually, such kind of formula has been used by Ulitsky and Elber in 1990 [3]. They use the formula to search the steepest descent path (SDP) from the transition state to the reactant or the product on the potential energy surface. Here the formula in Ref.[3] is applied to the free energy surface.

As we can see, one important point in the optimization of the path is calculating the free energy gradients. In practice, these gradients can be calculated by the constrained dynamics method [18–25]. The number of the constraints equals the number of the collective variables. In 2003, Schlitter and Klahn proved that the free energy function in the constrained simulation can be described by a concise formula [19]

$$\begin{array}{ll} F_{0\to 1} & =\int_{0}^{1}{\langle \frac{\partial H_{c}}{\partial \xi }\rangle }_{\xi }d\xi -{k_{B}T\text{ln}\langle {\left|\text{Z}\right|}^{-1/2}\rangle |}_{0}^{1}\\ & =\int_{0}^{1}{\langle \lambda \rangle }_{\xi }d\xi -{k_{B}T\text{ln}\langle {\left|\text{Z}\right|}^{-1/2}\rangle |}_{0}^{1} \end{array}$$ (6)

Here the variable *ξ* is the reaction coordinate that indicates the fraction of the snapshots encompassed by the path. It is also a function of the collective variables. *ξ* = 0 and *ξ* = 1 represent the initial and the final state respectively. *H*_*c*_ is the Hamiltonian function of the constrained system. *T* is temperature and *k*_*B*_ is the Boltzmann factor. <**λ**>_*ξ*_ represents the ensemble average of the *Lagrange* multiplier at *ξ* in the constrained simulation [51]. The last quantity ***Z*** is a *n*-dimensional matrix with the following element

$$Z_{\alpha \beta }=\sum_{i}^{}\frac{1}{m_{i}}\frac{\partial \sigma_{\alpha }}{\partial x_{i}}\frac{\partial \sigma_{\beta }}{\partial x_{i}}$$ (7)

*σ*_*α*_ and *σ*_*β*_ are the constraint equations and *m*_*i*_ is the mass of the *i*th atom in the related constraint [19]. By the formula, one can calculate the complete free energy profile on a path or a multidimensional surface that is defined by a set of collective variables [59].

From Eq 6, the gradient of the free energy function with respect to the reaction coordinate or the collective variables can also be calculated. It is clear that the free energy function in Eq 6 has two terms, and so does its derivative. However, as we tested before [26, 27], the second term is much smaller than the first term. So in this work, only the first term, i.e., the ensemble average of the *Lagrange* multiplier <**λ**>, is used in the calculation of the free energy gradient in Eqs 2 and 5. But, when calculating the free energy profile of the path, both terms are in use. With the free energy gradient, we can build a path gradually from the reactant to the product on the free energy landscape by the growing method (Eq 1) and optimize the path by the constrained dynamics method [19] at the same time (Eqs 3 and 5).

To monitor the convergence of the optimization, an error function is defined as follows. It is the average size of the free energy gradients of all the snapshots perpendicular to the tangent of the path

$$\sigma_{⊥}=\sum_{i=1}^{m}\frac{{\text{P}}_{op}^{\left(i\right)}\cdot {\text{P}}_{op}^{\left(i\right)}}{n\cdot m}$$ (8)

Here *n* and *m* are the number of the collective variables and the snapshots on the path, respectively. Smaller *σ*_⊥_ means the free energy gradients on the path are more parallel to the tangent of the path. And thus the path is more optimal on the free energy surface.

To show the performance of the presented method, we select two models in the calculation. The first is the ALA dipeptide with the sequence ACE-ALA-NME. Many simulations find that the peptide has two stable states in vacuum, named C7_eq_ and C7_ax_ [11, 29, 60–62]. In this work, the state C7_eq_ is set as the reactant and the state C7_ax_ is set as the product. Their backbone angles, Φ and Ψ, are chosen to be the collective variables. So the path exists in a two-dimensional collective variable space. Low complexity of the system allows us to do a sufficient sampling in the space for the test of the growing method in depth.

The second model is the 10-ALA peptide. It is well known that Alanine-rich peptides prefer the helical conformations in the aqueous environment [63–67], especially the *α*-helix. So here we set the *π*-helix as the reactant and the *α*-helix as the product. The main difference between these two secondary structures is the way to form hydrogen bonds. In *π*-helix, the hydrogen bonds are formed between the N-H group of an amino acid and the C = O group of the other at five residues earlier (*i*+5→*i*). And in *α*-helix, the interval between the hydrogen-bonded residues is 4 (*i*+4→*i*). In this application, we build a path from the *π*-helix to the *α*-helix by the growing method. Totally 12 collective variables are defined for the molecule, including one distance between the C_*α*_-atoms in the first and the last residue, five *i*+5→*i* hydrogen bonds in the standard *π*-helix structure, and six *i*+4→*i* hydrogen bonds in the standard *α*-helix structure. So many collective variables increase the complexity of the optimization problem. We want to find out if the presented method can work well in such a high-dimensional collective variable space.

The presented method has been written in Fortran code and inserted into the Tinker software [68]. All the simulations are performed at 298 K with AMBER PARM96 force field [69]. The positions and velocities of the atoms are updated by the Beeman's algorithm [70, 71] with the 1.0 fs time step. Berendsen method [72] controls the temperature. For the two models, ALA dipeptide is simulated in vacuum, 10-ALA peptide is simulated in the Generalized Born/Surface Area (GB/SA) implicit solvent [73, 74]. To accelerate the optimization, our code is parallelized by MPICH2 [75]. According to the number of the CPUs in the computer, all the snapshots on the path are evenly divided into parts for independent simulations.

## Results and discussion

**Fig 1:**
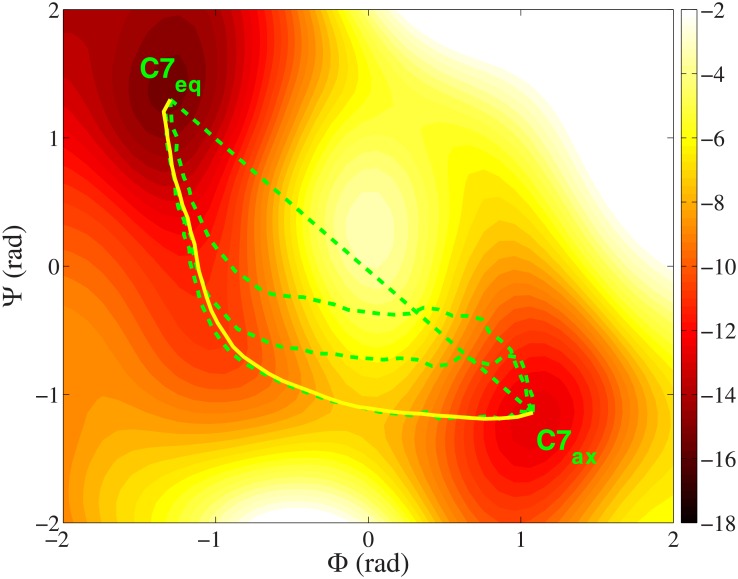
Different optimized paths of the ALA dipeptide between the reactant (C7_eq_) and the product (C7_ax_) in the two-dimensional collective variable space. The green dashed lines from up to down are the paths at 0, 50, 100 and 200 iterations by the interpolation method. The yellow solid line is the path generated by the growing method in this work. The unit of the free energy surface is kcal/mol.

The simulation results of the ALA dipeptide are shown in Fig 1. For a better illustration, the complete free energy landscape of the molecule in the whole collective variable space is also shown in the figure, which is calculated by adaptively biased molecular dynamics method (ABMD) [56–58] in a 500 ns trajectory (flooding time is 500 ps). The two free energy minima, reactant state C7_eq_ and product state C7_ax_, are marked in the figure too. The straight dashed line from C7_eq_ to C7_ax_ is the initial path built by the linear interpolation method. It is constituted of 40 evenly distributed snapshots. The free energy landscape in the figure shows that the interpolated path passes a high free energy barrier at the center, which requires to be optimized further. The green dashed lines from up to down in the figure are the optimized paths from the initial straight path by Eqs 3 and 5 at 0, 50, 100 and 200 iterations (step size *s*_*op*_ = 0.002). To calculate the free energy gradient (ensemble average of the *Lagrange* multiplier **λ** in Eq 6), each snapshot is simulated for 3 ps at each iteration step. From now on, the optimization method that starts from the interpolated path will be referred to as the "interpolation method" for short. As shown in the figure, the path moves away from the central free energy barrier gradually in the optimization and converges to the valley below. This reflects the ability of the interpolation method. However, performing simulations for all the snapshots on the path at every iteration step is time consuming. The whole optimization takes 200×40×0.003 ns = 24 ns (6.0×10^5^ MD steps per snapshot). If the number of the collective variables and the number of the snapshots increase further, this optimization process will be more slower.

Now we use the growing method to build and optimize the path (Eq 1). Different from the above interpolation method, our method starts from the single reactant state, not the whole interpolated path. The growth step size *s*_*g*_ in Eq 1 is 0.102. The weighing factor *w* in Eq 2 is 10.0 (in the test, the optimization with these parameters produces the growth path with the same number of snapshots as previous straight interpolated path). To ensure the smoothness of the path, all the existing snapshots are optimized for 2 steps by Eqs 3 and 5 when a new snapshot is grown. The optimizing step size *s*_*op*_ is 0.002, the same as before. Finally 40 snapshots are grown out of the path. The growth path is plotted by the yellow solid line in Fig 1. The shape of the path is in agreement with the previous optimized path by the interpolation method. And more importantly, the total simulation time is only 2×(40+40×(40–1)/2)×0.003 ns = 4.92 ns (1.23×10^5^ MD steps per snapshot). This is much shorter than the above interpolation method (6.0×10^5^ MD steps per snapshot). Clearly it is because that the growth path is shorter than the interpolation path in most time of the simulation. Fewer snapshots on the path will not only decrease the required simulation time at every iteration step, but also decrease the probability to pass the high free energy states at the early stage of the optimization.

**Fig 2:**
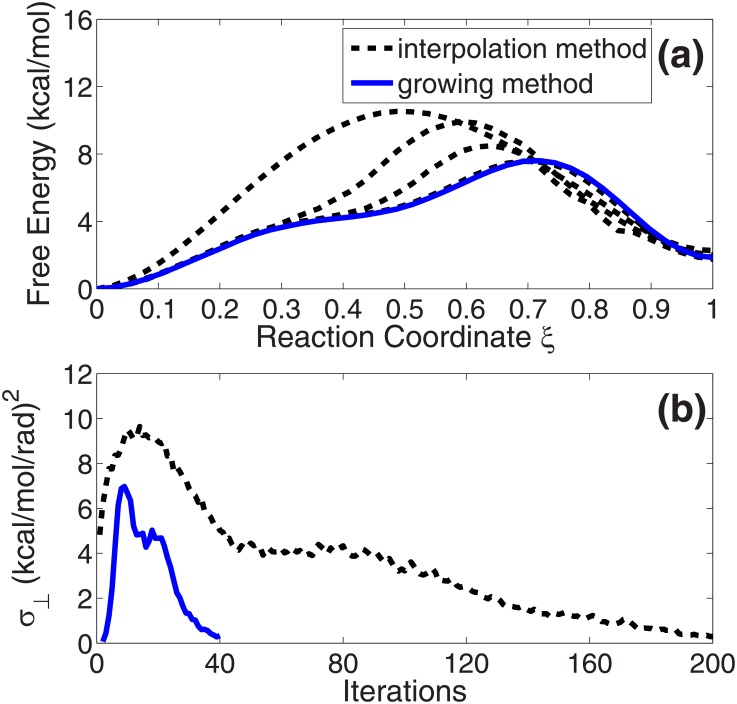
(a) Free energy profiles of ALA dipeptide on the optimized paths by the interpolation method at 0, 50, 100 and 200 iterations (black dashed lines from up to down) and the final growth path by the growing method (blue solid line). (b) The changes of the average free energy gradients perpendicular to the tangent of the path (*σ*_⊥_ in Eq 8) in the two optimizations.

Fig 2(a) shows the free energy profiles on the optimized paths by the interpolation method at 0, 50, 100 and 200 iterations (black dashed lines from up to down) and the path by the growing method (blue solid line). The free energies are calculated by Eq 6 [26]. The reaction coordinate *ξ* on the *x*-axis represents the location on the path. *ξ* = 0 is the reactant state (C7_eq_) and *ξ* = 1 is the product state (C7_ax_). Fig 2(b) gives the changes of the average free energy gradients perpendicular to the tangent of the path (*σ*_⊥_ in Eq 8) in the two optimizations. This is a parameter to show the convergence of the optimization. From the figure, we find that the end-to-end free energy differences on the path by the two methods are identical to each other. The product state is about 2.0 kcal/mol higher than the reactant state in both optimizations. This confirms the fact that the computed free energy is a state function, as it should be. The free energy difference between the states is independent of the transition path in the collective variable space. Moreover, the free energy profile of the path from the growing method is the same as the optimized path by the interpolation method at 200 iterations. This is reasonable because these two paths have the same shapes in Fig 1.

**Fig 3:**
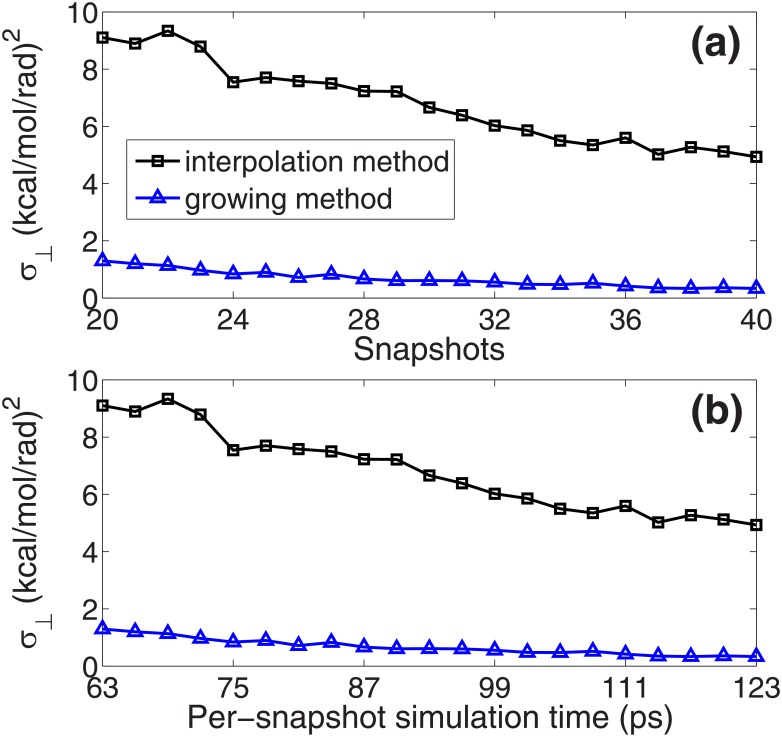
(a) Average perpendicular free energy gradients of the paths (*σ*_⊥_ in Eq 8) with different number of snapshots (from 20 to 40). The data in the optimization by the interpolation method are represented by the black squares and the data of the growing method are represented by the blue triangles. Note that the per-snapshot simulation time and the total simulation time are set to be the same for each path in the two optimizations (shown in (b)).

Fast convergence speed indicates that the growing method is quite efficient for the optimization of the path on the free energy surface. To compare the methods in depth, we perform different optimizations with different number of snapshots (from 20 to 40) by the two methods (the calculation quantities are the same). The final average perpendicular free energy gradients of the paths (*σ*_⊥_ in Eq 8) in the two optimizations are shown in Fig 3(a), and their per-snapshot simulation time are in Fig 3(b). The results show that the growing method has good performances for different paths with different lengths. The fast convergence speed of the method does not depend on the length of the path.

**Fig 4:**
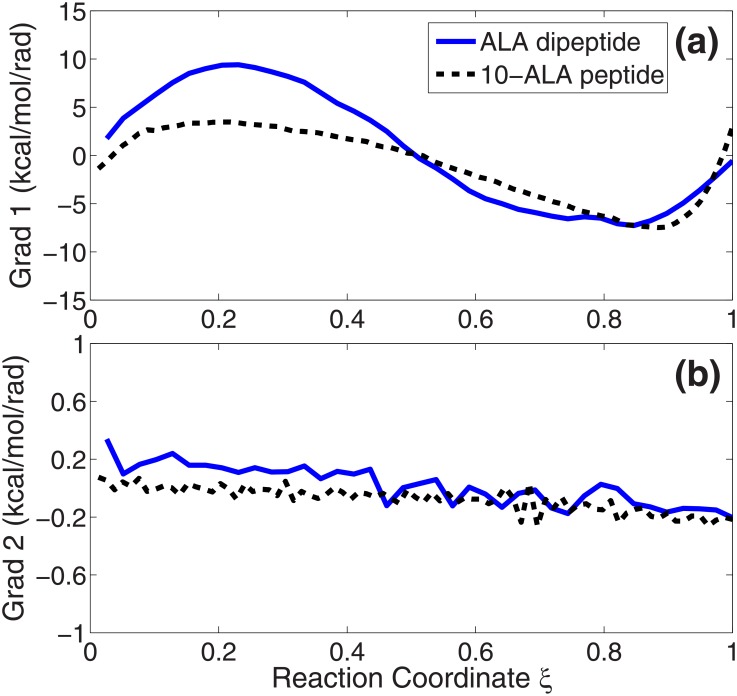
Two components of the free energy gradient of ALA dipeptide (blue solid lines) and 10-ALA dipeptide (black dashed lines) along the straight path from the reactant to the product. (a) The first term of the free energy gradient. (b) The second term of the free energy gradient.

In the following, we will discuss four important aspects on the path optimization problem. The first is the calculation of the free energy gradient for the path. Eq 6 shows that the free energy of each snapshot in the collective variable space has two terms. So the free energy gradient also has two terms. The first term comes from the constraint force in the simulation (ensemble average of the *Lagrange* multiplier) and the second is the correction term for the entropy loss from the unconstrained system to the constrained system [18, 19]. Due to the determinant of the metric tensor in Eq 6, analytic derivation of the second term is very complicated. For simple molecules like ALA dipeptide in the low dimensional collective variable space, it can be approximately calculated by the numerical central difference method [27]. However, for large molecules with lots of constraints, like 10-ALA in this work, even the numerical method becomes impractical. But we want to note that it is still possible to get the two components of the free energy gradient along the tangent of the path. The data can help us to evaluate the different weights of the two components. In Fig 4(a) and 4(b), we plot the two free energy gradient terms along the straight path from the reactant to the product for ALA dipeptide (blue solid lines) and 10-ALA peptide (black dashed lines), respectively. The results show that the second term is much smaller than the first term. For these two molecules, it is safe to use the first free energy gradient to update the shape of the path in the collective variable space.

**Fig 5:**
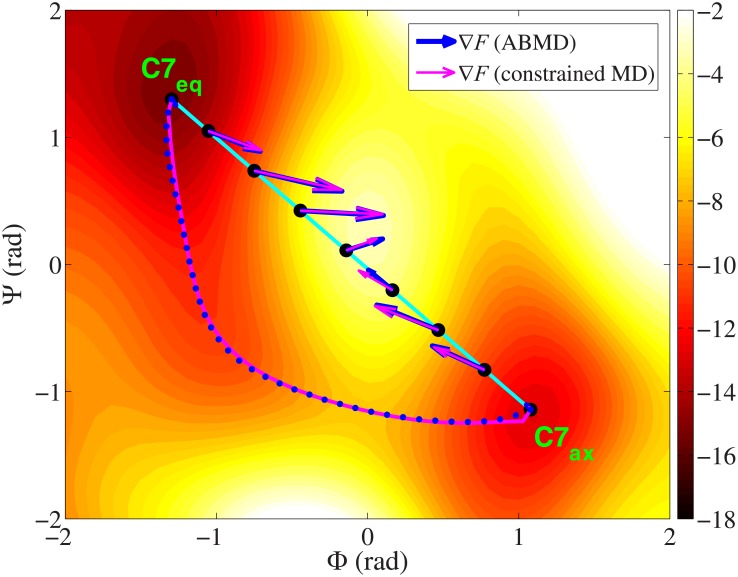
Selected free energy gradients of ALA dipeptide on a straight path. Blue arrows are the gradients ∇*F* from one prepared complete free energy surface in a 500 ns ABMD simulation [56–58] (multiplied by 0.06). Magenta arrows are the gradients ∇*F* from a few independent constrained simulations [18, 19] (multiplied by 0.06). The blue dotted curve and the magenta solid curve are the final optimized paths by the two kinds of free energy gradients, respectively. The unit of the free energy surface is kcal/mol. See the details on the optimizations in the main text.

The second aspect is the choice of the way to perform the path optimization. With some kinds of artificial potentials, a complete free energy surface in the whole collective variable space can be built by many well-known free energy calculation methods like ABMD [56–58] or Metadynamics [52–54]. So, before the path optimization, one can first prepare a complete free energy surface in a long simulation and then do the path optimization on this surface continuously. Otherwise, as in this work, one can calculate the free energy gradients during the optimization at every iteration step. When the path is updated, all the free energy gradients on the path are required to be calculated again in the new constrained simulations [18, 19]. In Fig 5, we show that these two ways of optimization are equivalent to each other for ALA dipeptide. The blue arrows on the straight path are the selected free energy gradients ∇*F* from the complete free energy surface by a 500 ns ABMD simulation [56–58] (multiplied by 0.06). The magenta arrows are the gradients ∇*F* on the current path obtained from a few independent constrained simulations [18, 19] (100 ps for each snapshot, multiplied by 0.06). It shows that the former free energy gradients are rather close to the latter. The final paths of the two optimizations are also in good agreement with each other (the blue dotted curve is from the former optimization on the complete surface and the magenta solid curve is from the latter, see the descriptions on the optimizations below). It is known that the optimization of the path on the free energy surface requires some MD simulations. But, performing the simulations before or during the optimization does not change the final results.

The third aspect we want to discuss is the acceleration methods in the path optimization. As introduced before, steepest descent method in Eq 3 is simple and practical. But the optimization is sensitive to the step size *s*_*op*_. Large *s*_*op*_ could cause the path to form kinks and small *s*_*op*_ will slow down the convergence speed. So, a variable step size may be useful. Initially one can set a relatively large step size. At every iteration step, if the sum of the free energies of all the snapshots on the path is smaller than the previous iteration step, then the step size is reduced by half. Otherwise, it is reset to the initial value. Besides the steepest descent method, quasi-Newton method is also an efficient optimization method. It updates the Hessian matrix iteratively by a method like L-BFGS [6, 7]. Applications shows the method performs well in the structural optimizations of the molecules [6, 7] with the all-atom force field [68]. However, in the test of the path optimization problem, we find that L-BFGS [6, 7] moves the snapshots too far in the collective variable space at every iteration step. This could twist the path and fail the optimization. So, two more operations are executed to solve this problem. The first is to reduce the movement distance of each snapshot by a step size control parameter *s*_*step*_

$$\chi_{new}^{\left(i\right)}=\chi_{old}^{\left(i\right)}+s_{step}\left(\chi_{bfgs}^{\left(i\right)}-\chi_{old}^{\left(i\right)}\right)$$ (9)

Here **χ**(*i*) *old* and **χ**(*i*) *new* are the current and the new position of the *i*th snapshot in the collective variable space. And **χ**(*i*) *bfgs* is the temporary position updated by L-BFGS [6, 7]. To reduce the movement distance, the control parameter *s*_*step*_ must be smaller than 1.0. The second operation is to smooth the updated path at every iteration by a positive objective function [30].

$$f=\sum_{i}^{}{\left(\theta (\chi^{\left(i-1\right)},\chi^{\left(i\right)}{,\chi }^{\left(i+1\right)})-\pi \right)}^{2}$$ (10)

**χ**^(*i*-1)^, **χ**^(*i*)^ and **χ**^(*i*+1)^ are the positions of the *i*-1th, *i*th and *i*+1th snapshot. *θ*(**χ**^(*i*-1)^, **χ**^(*i*)^, **χ**^(*i*+1)^) is the bending angle between the successive vectors **χ**^(*i*-1)^−**χ**^(*i*)^ and **χ**^(*i*+1)^−**χ**^(*i*)^. To make the path smooth, the objective function is optimized by the steepest descent method for 1000 steps with a step size 0.0001 (by default).

As we said before, all the three above optimization methods calculate the free energy gradients by some independent constrained simulations [18, 19] at every iteration step. Alternatively, one can also prepare a complete free energy surface in the collective variable space by ABMD [56–58] before the optimization. The surface data will be used later in the free energy gradient calculation and the path optimization.

**Fig 6:**
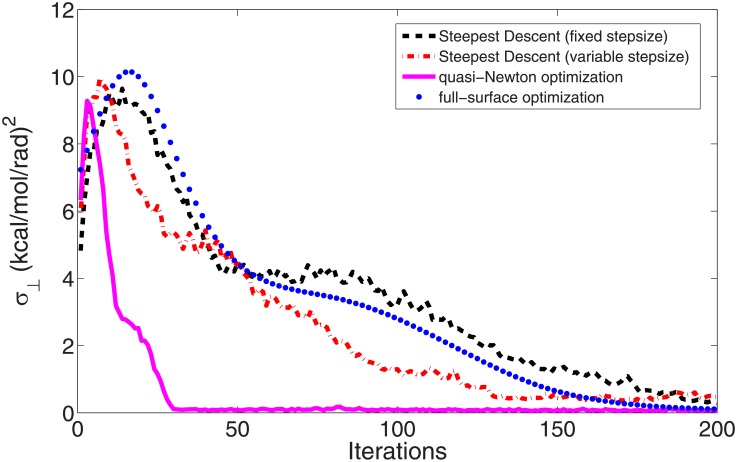
The changes of the average perpendicular free energy gradients (*σ*_⊥_ in Eq 8) in four different optimizations for ALA dipeptide from the straight path. The black dashed line is the data in the optimization by the steepest descent method with a fixed step size. The red dash-dotted line is the data of the steepest descent method with a variable step size. The magenta solid line is the data by the quasi-Newton method with L-BFGS formula [6, 7]. The blue dotted line is the data of the optimization on a prepared complete free energy surface from a ABMD simulation [56–58]. The shapes of the final optimized paths in the last two optimizations are plotted as the magenta solid curve and the blue dotted curve in Fig 5, respectively.

To compare the efficiencies, we perform four optimizations for ALA dipeptide from the straight path by the four different methods discussed above. The first is a steepest descent optimization with a fixed step size *s*_*op*_ = 0.002 (Eqs 3 and 5). The second is also a steepest descent optimization but with a variable step size (default step size *s*_*op*_ = 0.004). The third optimization is performed by Jorge Nocedal's L-BFGS code [6, 7] (step size control parameter *s*_*step*_ in Eq 9 is 0.2). To calculate the free energy gradients at every iteration step, one 3 ps constrained simulation [19] is launched for each snapshot at every iteration step. The last optimization is similar to the first one. It also uses the steepest descent method with a fixed step size 0.002. But the gradients are simply calculated from the complete free energy surface from a 500 ns ABMD simulation [56–58] (full-space optimization). The changes of the average perpendicular free energy gradients (*σ*_⊥_ in Eq 8) in all the four optimizations are shown in Fig 6. As we can see, quasi-Newton method with L-BFGS formula [6, 7] has the fastest convergence speed. The shape of the optimized path by the quasi-Newton method [6, 7] after 200 iterations is plotted as magenta solid curve in Fig 5. The path is perfectly overlapped with that from the last optimization on the complete free energy surface (blue dotted curve in Fig 5).

The last aspect is the choice of the driving force in the optimization. As shown in Eq 5, all the snapshots on the path are moved by their free energy forces (negative free energy gradients -∇*F*) in the collective variable space. Alternatively, as in ref. [3], they can also be moved by the energy forces -∇*V* too. Calculation of the free energy force (also called "thermodynamic mean force") demands more calculation time than the energy force. But the free energy force is corresponding to the driving force on the molecule from the free energy surface. With the free energy force, the molecule can search and locate the thermodynamically stable states reliably. And moreover, only the free energy force could provide the free energy profile on the path. The free energy profile is the key to the study of the reactions. So, unlike some previous methods that optimize the path by the energy force, in this work the presented method utilizes the free energy force in the optimization.

**Fig 7:**
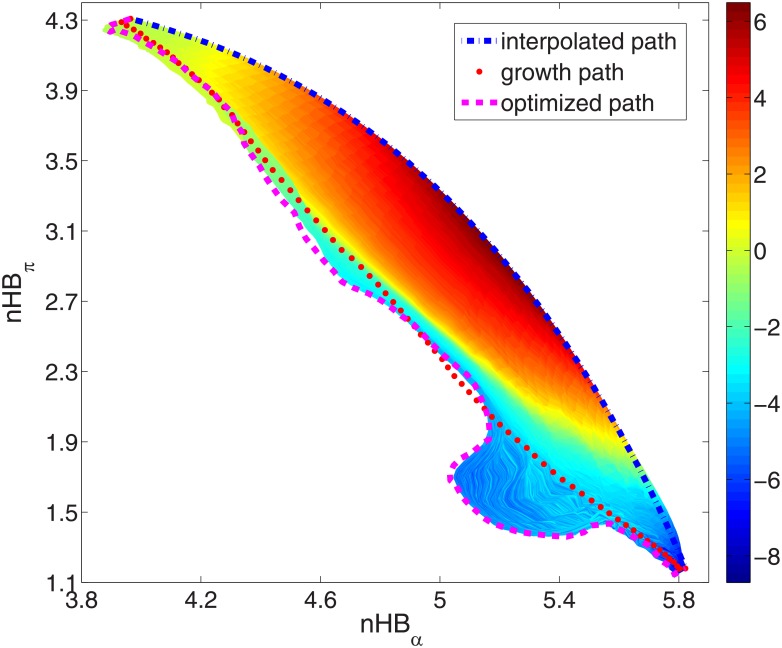
Projection of different paths of 10-ALA peptide from the reactant (*π*-helix, at the upper left) to the product (*α*-helix, at the bottom right) in a reduced two-dimensional space. The blue dash-dotted line, red dotted line and magenta dashed line represent the interpolated path, the growth path and the optimized path, respectively. The two collective variables, nHB_*α*_ and nHB_*π*_, are the effective numbers of the hydrogen bonds in the standard *α*-helix (*i*+4→*i*) and the standard *π*-helix (*i*+5→*i*) (Eq 11). The colors in the figure show the free energy profiles on the paths in the optimization from the interpolated path (unit: kcal/mol). Note that all the paths are originally defined and optimized in a 12-dimensional collective variable space.

Simulation results of ALA dipeptide show that the growing method works well in the low dimensional space. Now we turn to a more complicated model: 10-ALA peptide. It has ten residues and 103 atoms. 12 collective variables are defined for this model, including one end-to-end distance and 11 hydrogen bonds (five *i*+5→*i* hydrogen bonds in the standard *π*-helix structure, and six *i*+4→*i* hydrogen bonds in the standard *α*-helix structure). We select these collective variables is because that they are greatly different in the predefined reactant state (*π*-helix) and the product state (*α*-helix). Now we build a path by the growing method first. The growth step size *s*_*g*_ in Eq 1 is 0.1 and the weighting factor *w* in Eq 2 is 5.0. The path is optimized for 2 steps with the step size *s*_*op*_ = 0.001 after each new snapshot is grown. To calculate the free energy gradient, one 30 ps MD simulation is performed for each snapshot on the path. Finally the growth path has 80 snapshots. To show the path, we reduce the 12-dimensional collective variable space to a 2-dimensional space and project the growth path into the space (red dotted line in Fig 7). nHB_*α*_ on the *x*-axis is the effective number of the hydrogen bonds in the standard *α*-helix structure (*i*+4→*i*). And similarly, nHB_*π*_ is the effective number of the hydrogen bonds in the standard *π*-helix structure (*i*+5→*i*). Both collective variables are calculated by the formula in the users' manual of the AMBER software [76]. It defines one type of collective variable, N_OF_STRUCTURES, in the ABMD simulation [56–58]:

$$\text{nHB}=\sum_{i=1}^{k}\frac{1}{1+{\left(d/d_{0}\right)}^{6}}$$ (11)

Here the sum in the formula includes all the hydrogen bonds in the standard helical structure. So *k* = 6 for the *α*-helix and *k* = 5 for the *π*-helix. *d* is the actual bond length in the current structure and *d*_0_ is the reference bond length which is set to be 3.5 Å. For comparison, the projection of the linear interpolated path on the two-dimensional plane between the reactant and the product is also plotted in the figure (blue dash-dotted line). It can be found that the two paths are greatly different from each other. Although sharing the same reactant and the product, the growth path more prefers the intermediate snapshots with fewer hydrogen bonds than the interpolated path. To find out which one is more optimal on the free energy surface, we perform the optimizations for the paths and calculate the free energy profiles on them.

**Fig 8:**
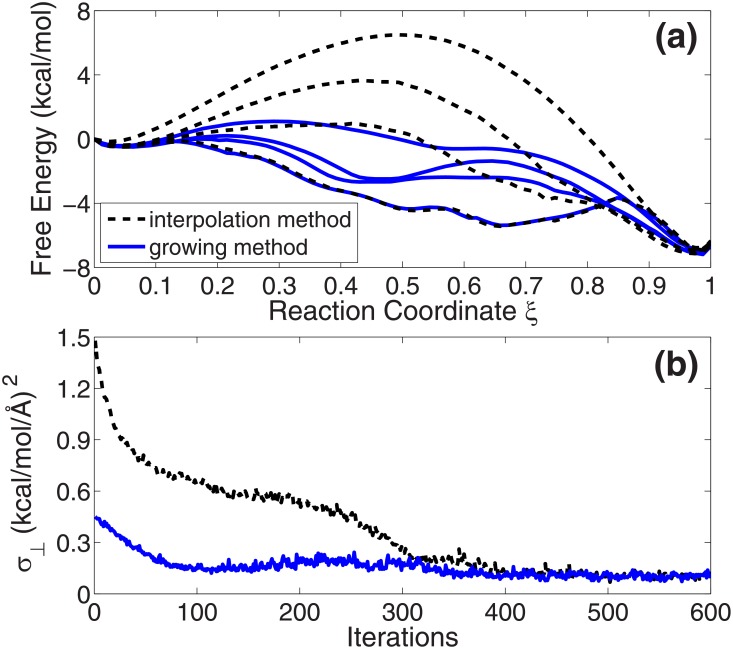
(a) Free energy profiles of 10-ALA peptide in the optimization from the interpolated path at 0, 100, 200 and 600 iterations (black dashed lines from up to down). And the free energy profiles of the paths optimized from the growth path at 0, 100, 200, and 600 iterations (blue solid lines from up to down). (b) The changes of the average free energy gradients perpendicular to the tangent of the path (*σ*_⊥_ in Eq 8) in the two optimizations.

Both of the two paths of 10-ALA peptide are further optimized for 600 iterations by quasi-Newton method with L-BFGS formula [6, 7]. Here the step size control parameter *s*_*step*_ in Eq 9 is 0.06. In the optimization from the interpolated path, all the evolved paths at the iterations are plotted in Fig 7. The colors in the figure represent the free energy values on the paths. They approximately reveal the original free energy landscape in the high dimensional collective variable space, from the initial interpolated path (blue dash-dotted line) to the final optimized path at 600 iterations (magenta dashed line). The free energy profiles of the paths at 0, 100, 200 and 600 iterations are plotted by black dashed lines from up to down in Fig 8(a). The same data of the optimization from the growth path are shown by blue solid lines. We find that the two optimizations produce the same results. Fig 8(b) gives the changes of the average perpendicular free energy gradients (*σ*_⊥_ in Eq 8) in the two optimizations. It indicates that the optimization from the path generated by the growing method converges much faster than that from the interpolated path. This confirms the result in Fig 7 that the initial position of the growth path on the free energy landscape (red dotted line) is closer to the final optimal path (magenta dashed line) than the interpolation path (blue dash-dotted line).

Moreover, there is a free energy minimum exists on the final optimized path. By checking the hydrogen bonds in the structure (the distance between backbone oxygen atom and hydrogen atom is smaller than 2.5 Å), we find that the snapshot at this minimum maintains the first five hydrogen bonds in the standard *α*-helix and the last hydrogen bond in the standard *π*-helix. So the *π*-to-*α* reaction can be divided into two stages approximately. At the first stage, the peptide breaks the first four *i*+5→*i* hydrogen bonds at the N-terminus of the *π*-helix and forms the first five *i*+4→*i* hydrogen bonds. Then, at the second stage, it breaks the last *i*+5→*i* hydrogen bond at the C-terminus and forms the remaining one *i*+4→*i* hydrogen bond at the same time. So, based on the findings from the simulation with AMBER PARM96 force field [69], it may be concluded that the *π*-to-*α* transition process of 10-ALA peptide starts from N-terminus and ends at C-terminus.

## Conclusions

Optimization of the path from the reactant to the product on the free energy surface is an interesting but also challenging problem. From the optimal path, one can know the reaction process in detail and conclude the reaction mechanism. In the study, building a good initial path is an important part of a successful optimization. The path can be built by a linear or higher order interpolation in the collective variable space [3, 4]. But such a geometric method does not consider the potential energy or the free energy of the molecule. If any part of the interpolated path passes the high free energy states at the beginning, it will take many additional iteration steps to move the path away from the states. Furthermore, at every iteration step, all the snapshots on the path must be simulated by the molecular dynamics method [70] for a period of time. Of course, this is also a time consuming process.

In this paper, we present a growing method to accelerate the construction and the optimization process of the path. This method uses the free energy gradients from the constrained simulations [18–25]. It grows the path gradually from the reactant to the product and optimizes the existing path constantly in the growing time. So the path can adaptively avoid the high free energy states at an early stage of the optimization and save much simulation time in the later iterations. Applications on two peptides show that the presented method is quite faster than the standard interpolation method. Actually, the growing strategy is not only useful for the construction of the path over the barriers (transition states), but also useful for the path that passes the basins on the free energy surface (intermediate states).

We want to note that the growing method in this work is a deterministic method. It always evolves to one final optimal path at the end of the optimization. For simple free energy landscape with one unique transition path between the reactant and the product, this method should work well. However, in a case of a molecule with multiple independent transition paths, it may not be so efficient. In such situations, one can utilize the "self-avoiding walk" strategy in discrete state model method [77, 78] or the "shooting" strategy in TPS method [43, 44]. These methods are able to generate lots of possible conformations or paths with weights in a quick way. Performing the optimization from these guess paths may save the simulation time. Moreover, the presented method calculates the free energy gradient of the snapshot on the path one by one. Each free energy gradient is determined by a structure ensemble corresponding to a set of collective variables. If there are some hidden barriers in the ensemble, insufficient conformational sampling in the simulation may produce the biased results. To improve the sampling, one can simulate a set of successive snapshots on the path simultaneously as in Ref. [79]. By a proper exchange between the snapshots according to their Hamiltonians, the molecule should have a high chance to pass the hidden barriers in the ensemble.

## Supporting information

S1 File It contains the molecular structures, the simulation parameters and the results in this paper. (ZIP)

## References

[1] Hu H, Lu Z, Yang W. QM/MM Minimum Free Energy Path: Methodology and Application to Triosephosphate Isomerase. J Chem Theory Comput. 2007;3:390–406. 10.1021/ct600240y 19079734 PMC2600730

[2] Del Campo JM, Koster AM. A hierarchical transition state search algorithm. J Chem Phys. 2008;129:024107 10.1063/1.2950083 18624516

[3] Ulitsky A, Elber R. A new technique to calculate steepest descent paths in flexible polyatomic systems. J Chem Phys. 1990;92:1510–1.

[4] Tyka MD, Clarke AR, Sessions RB. An Efficient, Path-Independent Method for Free-Energy Calculations. J Phys Chem B. 2006;110:17212–20. 10.1021/jp060734j 16928020

[5] Chen C, Xiao Y. Accurate free energy calculation along optimized paths. J Comput Chem. 2010;31:1368–75. 10.1002/jcc.21420 19859916

[6] Nocedal J. Updating Quasi-Newton Matrices with Limited Storage. Math Comput. 1980;35:773–82.

[7] Lui DC, Nocedal J. Limited memory BFGS method for large scale optimization. Mathematical Programming. 1989;45:503–28.

[8] Elber R, Karplus M. A method for determining reaction paths in large molecules: Application to myoglobin. Chem Phys Lett. 1987;139:375–80.

[9] Henkelman G, Jonsson H. Improved tangent estimate in the nudged elastic band method for finding minimum energy paths and saddle points. J Chem Phys. 2000;113:9978–85.

[10] Brokaw JB, Haas KR, Chu JW. Reaction Path Optimization with Holonomic Constraints and Kinetic Energy Potentials. J Chem Theory Comput. 2009;5:2050–61. 10.1021/ct9001398 26613147

[11] Maragliano L, Fischer A, Vanden-Eijnden E, Ciccotti G. String method in collective variables: Minimum free energy paths and isocommittor surfaces. J Chem Phys. 2006;125:024106. 10.1063/1.2212942 16848576

[12] Maragliano L, Vanden-Eijnden E. On-the-fly string method for minimum free energy paths calculation. Chem Phys Lett. 2007;446:182–90.

[13] Rosta E, Nowotny M, Yang W, Hummer G. Catalytic Mechanism of RNA Backbone Cleavage by Ribonuclease H from Quantum Mechanics/Molecular Mechanics Simulations. J Am Chem Soc. 2011;133(23):8934–41. 10.1021/ja200173a 21539371 PMC3110985

[14] Ovchinnikov V, Karplus M, Vanden-Eijnden E. Free energy of conformational transition paths in biomolecules: The string method and its application to myosin VI. J Chem Phys. 2011;134(8):085103 10.1063/1.3544209 21361558 PMC3060930

[15] Ren W, Vanden-Eijnden E. A climbing string method for saddle point search. J Chem Phys. 2013;138:134105 10.1063/1.4798344 23574206

[16] Behn A, Zimmerman PM, Bell AT, Head-Gordon M. Efficient exploration of reaction paths via a freezing string method. J Chem Phys. 2011;135:224108 10.1063/1.3664901 22168681

[17] Sharada SM, Zimmerman PM, Bell AT, Head-Gordon M. Automated Transition State Searches without Evaluating the Hessian. J Chem Theory Comput. 2012;8:5166–74. 10.1021/ct300659d 26593206

[18] Schlitter J, Klahn M. A new concise expression for the free energy of a reaction coordinate. J Chem Phys. 2003;118:2057–60.

[19] Schlitter J, Klahn M. The free energy of a reaction coordinate at multiple constraints: a concise formulation. Mol Phys. 2003;101:3439–43.

[20] Schlitter J. Constraint methods for determining pathways and free energy of activated processes. Eur Phys J Spec Top. 2011;200: 91–105.

[21] Carter EA, Ciccotti G, Hynes JT, Kapral R. Constrained reaction coordinate dynamics for the simulation of rare events. Chem Phys Lett. 1989;156:472–7.

[22] Sprik M, Ciccotti G. Free energy from constrained molecular dynamics. J Chem Phys. 1998;109:7737–44.

[23] Coluzza I, Sprik M, Ciccotti G. Constrained reaction coordinate dynamics for systems with constraints. Mol Phys. 2003;101:2885–94.

[24] Ciccotti G, Kapral R, Vanden-Eijnden E. Blue moon sampling, vectorial reaction coordinates, and unbiased constrained dynamics. Chemphyschem. 2005;6:1809–14. 10.1002/cphc.200400669 16144000

[25] den Otter WK. Thermodynamic integration of the free energy along a reaction coordinate in Cartesian coordinates. J Chem Phys. 2000;112:7283–92.

[26] Chen C, Huang Y, Xiao Y. Free-energy calculations along a high-dimensional fragmented path with constrained dynamics. Phys Rev E. 2012;86:031901. 10.1103/PhysRevE.86.031901 23030938

[27] Chen C, Huang Y, Ji X, Xiao Y. Efficiently finding the minimum free energy path from steepest descent path. J Chem Phys. 2013;138:164122 10.1063/1.4799236 23635126

[28] Pan AC, Sezer D, Roux B. Finding transition pathways using the string method with swarms of trajectories. J Phys Chem B. 2008;112:3432–40. 10.1021/jp0777059 18290641 PMC2757167

[29] Leines GD, Ensing B. Path Finding on High-Dimensional Free Energy Landscapes. Phys Rev Lett. 2012;109(2):020601 10.1103/PhysRevLett.109.020601 23030145

[30] Chen C, Huang Y, Jiang W, Xiao Y. A fast tomographic method for searching the minimum free energy path. J Chem Phys. 2014;141:154109 10.1063/1.4897983 25338883

[31] Chen C. Calculation of the local free energy landscape in the restricted region by the modified tomographic method. J Phys Chem B. 2016;120:3061–71. 10.1021/acs.jpcb.5b11892 26974860

[32] Peters B, Heyden A, Bell AT, Chakraborty A. A growing string method for determining transition states: Comparison to the nudged elastic band and string methods. J Chem Phys. 2004;120:7877–86. 10.1063/1.1691018 15267702

[33] Behn A, Zimmerman PM, Bell AT, Head-Gordon M. Incorporating Linear Synchronous Transit Interpolation into the Growing String Method: Algorithm and Applications. J Chem Theory Comput. 2011;7:4019–25. 10.1021/ct200654u 26598348

[34] Zimmerman PM. Growing string method with interpolation and optimization in internal coordinates: Method and examples. J Chem Phys. 2013;138:184102 10.1063/1.4804162 23676024

[35] Zimmerman P. Reliable Transition State Searches Integrated with the Growing String Method. J Chem Theory Comput. 2013;9:3043–50. 10.1021/ct400319w 26583985

[36] Quapp W. A growing string method for the reaction pathway defined by a Newton trajectory. J Chem Phys. 2005;122:174106 10.1063/1.1885467 15910022

[37] Zimmerman PM. Single-Ended Transition State Finding with the Growing String Method. J Comput Chem. 2015;36:601–11. 10.1002/jcc.23833 25581279

[38] Goodrow A, Bell AT, Head-Gordon M. Development and application of a hybrid method involving interpolation and ab initio calculations for the determination of transition states. J Chem Phys. 2008;129:174109 10.1063/1.2992618 19045335

[39] Goodrow A, Bell AT, Head-Gordon M. Transition state-finding strategies for use with the growing string method. J Chem Phys. 2009;130:244108 10.1063/1.3156312 19566143

[40] Fleurat-Lessard P, Ziegler T. Tracing the minimum-energy path on the free-energy surface. J Chem Phys. 2005;123:084101 10.1063/1.1948367 16164276

[41] Maeda S, Ohno K, Morokuma K. Systematic exploration of the mechanism of chemical reactions: the global reaction route mapping (GRRM) strategy using the ADDF and AFIR methods. Phys Chem Chem Phys. 2013;15:3683–701. 10.1039/c3cp44063j 23389653

[42] Pietrucci F, Saitta AM. Formamide reaction network in gas phase and solution via a unified theoretical approach: Toward a reconciliation of different prebiotic scenarios. Proc Natl Acad Sci USA. 2015;112:15030–5. 10.1073/pnas.1512486112 26598679 PMC4679036

[43] Dellago C, Bolhuis PG, Csajka FS, Chandler D. Transition path sampling and the calculation of rate constants. J Chem Phys. 1998;108:1964–77.

[44] Bolhuis PG, Dellago C, Geissler PL, Chandler D. Transition Path Sampling: throwing ropes over through mountain passes, in the dark. Ann Rev Phys Chem. 2002;53:291–318. 11972010 10.1146/annurev.physchem.53.082301.113146

[45] Park S, Khalili-Araghi F, Tajkhorshid E, Schulten K. Free energy calculation from steered molecular dynamics simulations using Jarzynski's equality. J Chem Phys. 2003;119:3559–66.

[46] Perisic O, Lu H. On the improvement of free-energy calculation from steered molecular dynamics simulations using adaptive stochastic perturbation protocols. Plos One. 2014;9:e101810 10.1371/journal.pone.0101810 25232859 PMC4169427

[47] Schlitter J, Engels M, Kruger P, Jacoby E, Wollmer A. Targeted molecular dynamics simulation of conformational change-application to the T↔R transition in insulin. Mol Sim. 1993;10:291–308.

[48] Schlitter J, Engels M, Kruger P. Targeted molecular dynamics: a new approach for searching pathways of conformational transitions. J Mol Graph. 1994;12:84–9. 7918256 10.1016/0263-7855(94)80072-3

[49] Matsunaga Y, Fujisaki H, Terada T, Furuta T, Moritsugu K, Kidera A.. Minimum Free Energy Path of Ligand-Induced Transition in Adenylate Kinase. PLoS Comput Biol 2012;8:e1002555 10.1371/journal.pcbi.1002555 22685395 PMC3369945

[50] Moradi M, Sagui C, Roland C. Calculating relative transition rates with driven nonequilibrium simulations. Chem Phys Lett. 2011;518:109–13.

[51] Ryckaert JP, Ciccotti G, Berendsen HJC. Numerical Integration of the Cartesian Equations of Motion of a System with Constraints: Molecular Dynamics of n-Alkanes. J Comp Phys. 1977;23:327–41.

[52] Laio A, Parrinello M. Escaping free-energy minima. Proc Natl Acad Sci USA. 2002;99(20):12562–6 10.1073/pnas.202427399 12271136 PMC130499

[53] Laio A, Rodriguez-Fortea A, Gervasio FL, Ceccarelli M, Parrinello M. Assessing the Accuracy of Metadynamics. J Phys Chem B. 2005;109:6714–21. 10.1021/jp045424k 16851755

[54] Bussi G, Laio A, Parrinello M. Equilibrium free energies from nonequilibrium metadynamics. Phys Rev Lett. 2006;96:090601 10.1103/PhysRevLett.96.090601 16606249

[55] Bian Y, Zhang J, Wang J, Wang J, Wang W. Free energy landscape and multiple folding pathways of an H-type RNA pseudoknot. Plos One. 2015;10:e0129089 10.1371/journal.pone.0129089 26030098 PMC4451515

[56] Babin V, Roland C, Sagui C. Adaptively biased molecular dynamics for free energy calculations. J Chem Phys. 2008;128:134101 10.1063/1.2844595 18397047

[57] Moradi M, Babin V, Roland C, Darden TA, Sagui C. Conformations and free energy landscapes of polyproline peptides. Proc Natl Acad Sci USA. 2009;106:20746–51. 10.1073/pnas.0906500106 19923435 PMC2791577

[58] Babin V, Sagui C. Conformational free energies of methyl-alpha-L-iduronic and methyl-beta-D-glucuronic acids in water. J Chem Phys. 2010;132:104108 10.1063/1.3355621 20232948

[59] Li W, Rudack T, Gerwert K, Graäter F, Schlitter J. Exploring the multidimensional free energy surface of phosphoester hydrolysis with constrained QM/MM dynamics. J Chem Theory Comput. 2012;8:3596–604. 10.1021/ct300022m 26593005

[60] Apostolakis J, Ferrara P, Caflish A. Calculation of conformational transitions and barriers in solvated systems: Application to the alanine dipeptide in water. J Chem Phys. 1999;110:2099–108.

[61] Abrams JB, Tuckerman ME. Efficient and Direct Generation of Multidimensional Free Energy Surfaces via Adiabatic Dynamics without Coordinate Transformations. J Phys Chem B. 2008;112:15742–57. 10.1021/jp805039u 19367870

[62] Ensing B, Vivo MD, Liu Z, Moore P, Klein ML. Metadynamics as a Tool for Exploring Free Energy Landscapes of Chemical Reactions. Acc Chem Res. 2006;39:73–81. 10.1021/ar040198i 16489726

[63] Scholtz JM, Qian H, York EJ, Stewart JM, Baldwin RL. Parameters of helix-coil transition theory for alanine-based peptides of varying chain lengths in water. Biopolymers. 1991;31:1463–70. 10.1002/bip.360311304 1814498

[64] Baldwin RL. alpha-helix formation by peptides of defined sequence. Biophys Chem. 1995;55:127–35. 7632873 10.1016/0301-4622(94)00146-b

[65] Bousquet JA, Garbay C, Roques BP, Mely Y. Circular dichroic investigation of the native and non-native conformational states of the growth factor receptor-binding protein 2 N-terminal src homology domain 3: effect of binding to a proline-rich peptide from guanine nucleotide exchange factor. Biochemistry. 2000;39:7722–35. 10869177 10.1021/bi9929103

[66] Odaert B, Baleux F, Huynh-Dinh T, Neumann JM, Sanson A. Nonnative Capping Structure Initiates Helix Folding in an Annexin I Fragment. A 1H NMR Conformational Study. Biochemistry-Us. 1995;34:12820–9. 10.1021/bi00039a043 7548037

[67] Wu X, Wang S. Helix Folding of an Alanine-Based Peptide in Explicit Water. J Phys Chem B. 2001;105:2227–35.

[68] Ren P, Ponder JW. Polarizable Atomic Multipole Water Model for Molecular Mechanics Simulation. J Phys Chem B. 2003;107:5933–47.

[69] Cornell WD, Cieplak P, Bayly CI, Gould IR, Merz KMJ, Ferguson DM, et al A Second Generation Force Field for the Simulation of Proteins, Nucleic Acids, and Organic Molecules. J Am Chem Soc. 1995;117:5179–97.

[70] Beeman D. Some multistep methods for use in molecular dynamics calculations. J Comput Phys. 1976;20:130–9.

[71] Levitt M, Meirovitch H, Huber R. Integrating the equations of motion. J Mol Biol. 1983;168:617–20. 6193281 10.1016/s0022-2836(83)80305-2

[72] Berendsen HJC, Postma JPM, van Gunsteren WF, Dinola A, Haak JR. Molecular dynamics with Coupling to an External Bath. J Chem Phys. 1984;81:3684–90.

[73] Still VC, Tempezvk A, Hawley RC, Hendrickson T. Semianalytical treatment of solvation for molecular mechanics and dynamics. J Am Chem Soc. 1990;112:6127–9.

[74] Qiu D, Shenkin PS, Hollinger FP, Still WC. The GB/SA Continuum Model for Solvation. A Fast Analytical Method for the Calculation of Approximate Born Radii. J Phys Chem A. 1997;101:3005–14.

[75] Buntinas D, Mercier G, Gropp W. Implementation and Evaluation of Shared-Memory Communication and Synchronization Operations in MPICH2 using the Nemesis Communication Subsystem. Parallel Comput. 2007;33:634–44.

[76] Case DA, Darden TA, Cheatham III TE, Simmerling CL, Wang J, Duke RE, et al. AMBER 11. University of California, San Francisco. 2010.

[77] Zhang J, Lin M, Chen R, Wang W, Liang J. Discrete state model and accurate estimation of loop entropy of RNA secondary structures. J Chem Phys. 2008;128:125107 10.1063/1.2895050 18376982 PMC2494904

[78] Zhang J, Dundas J, Lin M, Chen R, Wang W, Liang J. Prediction of geometrically feasible three-dimensional structures of pseudoknotted RNA through free energy estimation. RNA. 2009;15:2248–63. 10.1261/rna.1723609 19864433 PMC2779689

[79] Rosta E, Yang W, Hummer G. Calcium Inhibition of Ribonuclease H1 Two-Metal Ion Catalysis. J Am Chem Soc. 2014;136:3137–44. 10.1021/ja411408x 24499076 PMC3985467

